# 2,5-Bis[(3-chloro­benz­yl)sulfan­yl]-1,3,4-thia­diazole

**DOI:** 10.1107/S1600536812019150

**Published:** 2012-05-05

**Authors:** Na-Bo Sun, Jian-Zhong Jin, Wei Ke

**Affiliations:** a College of Biology and Environmental Engineering, Zhejiang Shuren University, Hangzhou 310015, People’s Republic of China

## Abstract

The complete mol­ecule of the title compound, C_16_H_12_Cl_2_N_2_S_3_, is generated by crystallographic twofold symmetry, with the S atom of the thiadiazole ring lying on the rotation axis. The dihedral angle between the mean planes of the 1,3,4-thia­diazole and benzene rings is 87.19 (7)°. In the crystal, mol­ecules are linked by C—H⋯N inter­actions and short S⋯S contacts [3.3389 (9) Å] occur.

## Related literature

For details of the synthesis, see: Liu *et al.* (2012 ▶); Tan *et al.* (2012 ▶). For a related structure, see: Liu & Liu (2011 ▶. For the biological activity of related compounds, see: Liu *et al.* (2011*a* ▶,*b* ▶).
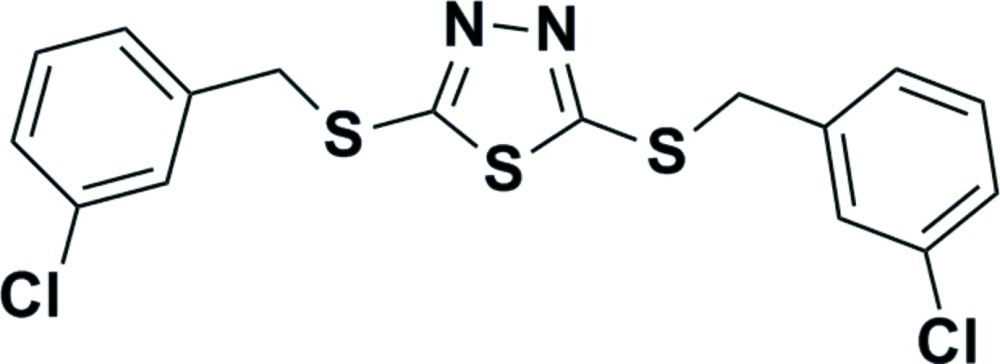


## Experimental

### 

#### Crystal data

C_16_H_12_Cl_2_N_2_S_3_
*M* *_r_* = 399.36
Monoclinic, 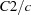

*a* = 17.200 (3) Å
*b* = 5.6604 (11) Å
*c* = 17.524 (4) Å
β = 92.56 (3)°
*V* = 1704.4 (6) Å^3^
*Z* = 4
Mo *K*α radiation
μ = 0.75 mm^−1^
*T* = 113 K
0.12 × 0.10 × 0.06 mm

#### Data collection

Rigaku Saturn CCD diffractometer
Absorption correction: multi-scan (*CrystalClear*; Rigaku/MSC, 2005 ▶) *T*_min_ = 0.916, *T*_max_ = 0.957
5343 measured reflections
1491 independent reflections
1382 reflections with *I* > 2σ(*I*)
*R*_int_ = 0.028

#### Refinement

*R*[*F* ^2^ > 2σ(*F* ^2^)] = 0.024
*wR*(*F* ^2^) = 0.063
*S* = 1.17
1491 reflections
105 parameters
H-atom parameters constrained
Δρ_max_ = 0.25 e Å^−3^
Δρ_min_ = −0.22 e Å^−3^

### 

Data collection: *CrystalClear* (Rigaku/MSC, 2005 ▶); cell refinement: *CrystalClear*; data reduction: *CrystalClear* (Rigaku/MSC, 2005 ▶); program(s) used to solve structure: *SHELXS97* (Sheldrick, 2008 ▶); program(s) used to refine structure: *SHELXL97* (Sheldrick, 2008 ▶); molecular graphics: *SHELXTL* (Sheldrick, 2008 ▶); software used to prepare material for publication: *SHELXTL*.

## Supplementary Material

Crystal structure: contains datablock(s) global, I. DOI: 10.1107/S1600536812019150/hb6758sup1.cif

Structure factors: contains datablock(s) I. DOI: 10.1107/S1600536812019150/hb6758Isup2.hkl

Supplementary material file. DOI: 10.1107/S1600536812019150/hb6758Isup3.cml

Additional supplementary materials:  crystallographic information; 3D view; checkCIF report

## Figures and Tables

**Table 1:** Hydrogen-bond geometry (Å, °)

*D*—H⋯*A*	*D*—H	H⋯*A*	*D*⋯*A*	*D*—H⋯*A*
C3—H3⋯N1^i^	0.93	2.59	3.514 (2)	172

Symmetry code: (i) 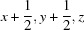.

**Fig. 1.**
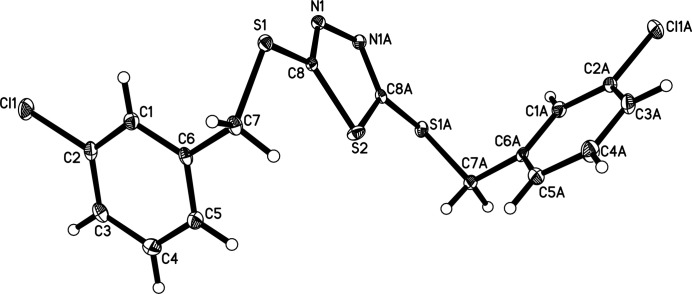
The molecular structure of (I). Displacement ellipsoids are drawn at the 30% probability level.

## supplementary crystallographic information

### Experimental

To a stirred solution of 1,3,4-thiadiazole-2,5-dithiol (5.1 mmol) and potassium carbonate (5.6 mmol) in DMF (15 ml), 1-chloro-3-(chloromethyl)benzene (5.6 mmol) was added dropwise. The resulting mixture was stirred at room temperature overnight. The mixture was poured into water, the precipitate formed was filtered off and recrystallized from acetone to give title compound in good yields. Compound was dissolved in hot alcohol and the resulting solution was allowed to stand in air at room temperature to colourless blocks of the title compound.

### Refinement

All the H atoms were positioned geometrically (C—H = 0.93–0.97 Å) and refined as riding with *U*_iso_(H) = 1.2*U*_eq_(C) or 1.5*U*_eq_(methyl C).

### Crystal data

**Table:**

C_16_H_12_Cl_2_N_2_S_3_	*F*(000) = 816
*M**_r_* = 399.36	*D*_x_ = 1.556 Mg m^−^^3^
Monoclinic, *C*2/*c*	Mo *K*α radiation, λ = 0.71073 Å
*a* = 17.200 (3) Å	Cell parameters from 2634 reflections
*b* = 5.6604 (11) Å	θ = 3.3–27.9°
*c* = 17.524 (4) Å	µ = 0.75 mm^−^^1^
β = 92.56 (3)°	*T* = 113 K
*V* = 1704.4 (6) Å^3^	Block, colorless
*Z* = 4	0.12 × 0.10 × 0.06 mm

### Data collection

**Table:**

Rigaku Saturn CCD diffractometer	1491 independent reflections
Radiation source: rotating anode	1382 reflections with *I* > 2σ(*I*)
Confocal monochromator	*R*_int_ = 0.028
ω scans	θ_max_ = 25.0°, θ_min_ = 3.3°
Absorption correction: multi-scan (*CrystalClear*; Rigaku/MSC, 2005)	*h* = −20→20
*T*_min_ = 0.916, *T*_max_ = 0.957	*k* = −6→6
5343 measured reflections	*l* = −14→20

### Refinement

**Table:**

Refinement on *F*^2^	Primary atom site location: structure-invariant direct methods
Least-squares matrix: full	Secondary atom site location: difference Fourier map
*R*[*F*^2^ > 2σ(*F*^2^)] = 0.024	Hydrogen site location: inferred from neighbouring sites
*wR*(*F*^2^) = 0.063	H-atom parameters constrained
*S* = 1.17	*w* = 1/[σ^2^(*F*_o_^2^) + (0.0254*P*)^2^ + 1.1171*P*] where *P* = (*F*_o_^2^ + 2*F*_c_^2^)/3
1491 reflections	(Δ/σ)_max_ = 0.001
105 parameters	Δρ_max_ = 0.25 e Å^−^^3^
0 restraints	Δρ_min_ = −0.22 e Å^−^^3^

### Special details

**Table:**

Geometry. All e.s.d.'s (except the e.s.d. in the dihedral angle between two l.s. planes) are estimated using the full covariance matrix. The cell e.s.d.'s are taken into account individually in the estimation of e.s.d.'s in distances, angles and torsion angles; correlations between e.s.d.'s in cell parameters are only used when they are defined by crystal symmetry. An approximate (isotropic) treatment of cell e.s.d.'s is used for estimating e.s.d.'s involving l.s. planes.
Refinement. Refinement of *F*^2^ against ALL reflections. The weighted *R*-factor *wR* and goodness of fit *S* are based on *F*^2^, conventional *R*-factors *R* are based on *F*, with *F* set to zero for negative *F*^2^. The threshold expression of *F*^2^ > σ(*F*^2^) is used only for calculating *R*-factors(gt) *etc*. and is not relevant to the choice of reflections for refinement. *R*-factors based on *F*^2^ are statistically about twice as large as those based on *F*, and *R*- factors based on ALL data will be even larger.

### Fractional atomic coordinates and isotropic or equivalent isotropic displacement parameters (Å^2^)

**Table:**

	*x*	*y*	*z*	*U*_iso_*/*U*_eq_	
S1	0.03445 (2)	−0.05340 (7)	0.41412 (2)	0.01769 (13)	
S2	0.0000	0.13382 (10)	0.2500	0.01979 (16)	
Cl1	0.36343 (2)	−0.14225 (8)	0.42335 (3)	0.03039 (14)	
N1	0.01164 (7)	−0.3031 (2)	0.28829 (7)	0.0161 (3)	
C1	0.22023 (9)	0.0583 (3)	0.41728 (9)	0.0198 (3)	
H1	0.2061	−0.0599	0.4508	0.024*	
C2	0.29495 (9)	0.0682 (3)	0.39189 (9)	0.0206 (4)	
C3	0.31795 (9)	0.2422 (3)	0.34242 (9)	0.0255 (4)	
H3	0.3687	0.2464	0.3263	0.031*	
C4	0.26429 (10)	0.4097 (3)	0.31736 (10)	0.0284 (4)	
H4	0.2789	0.5278	0.2840	0.034*	
C5	0.18844 (10)	0.4023 (3)	0.34188 (9)	0.0232 (4)	
H5	0.1525	0.5150	0.3245	0.028*	
C6	0.16605 (9)	0.2277 (3)	0.39205 (8)	0.0171 (3)	
C7	0.08449 (9)	0.2289 (3)	0.42062 (9)	0.0190 (3)	
H7A	0.0540	0.3448	0.3916	0.023*	
H7B	0.0869	0.2793	0.4736	0.023*	
C8	0.01873 (8)	−0.0905 (3)	0.31589 (8)	0.0136 (3)	

### Atomic displacement parameters (Å^2^)

**Table:**

	*U*^11^	*U*^22^	*U*^33^	*U*^12^	*U*^13^	*U*^23^
S1	0.0171 (2)	0.0229 (2)	0.0129 (2)	−0.00199 (16)	−0.00114 (15)	0.00075 (15)
S2	0.0314 (3)	0.0127 (3)	0.0149 (3)	0.000	−0.0042 (2)	0.000
Cl1	0.0197 (2)	0.0303 (3)	0.0404 (3)	0.00735 (18)	−0.00680 (18)	0.00062 (19)
N1	0.0158 (6)	0.0171 (7)	0.0153 (6)	−0.0004 (6)	0.0002 (5)	0.0007 (5)
C1	0.0191 (8)	0.0213 (8)	0.0187 (8)	−0.0008 (7)	−0.0024 (6)	−0.0009 (7)
C2	0.0169 (8)	0.0213 (8)	0.0230 (8)	0.0029 (7)	−0.0063 (6)	−0.0050 (7)
C3	0.0143 (8)	0.0314 (10)	0.0308 (9)	−0.0037 (7)	0.0006 (7)	−0.0027 (8)
C4	0.0230 (10)	0.0274 (10)	0.0348 (10)	−0.0044 (8)	0.0018 (7)	0.0074 (8)
C5	0.0198 (9)	0.0204 (9)	0.0289 (9)	0.0020 (7)	−0.0031 (7)	0.0004 (7)
C6	0.0153 (8)	0.0201 (8)	0.0156 (7)	−0.0020 (7)	−0.0030 (6)	−0.0070 (6)
C7	0.0166 (8)	0.0214 (8)	0.0190 (8)	−0.0002 (7)	−0.0009 (6)	−0.0061 (7)
C8	0.0096 (7)	0.0164 (8)	0.0149 (8)	0.0001 (6)	0.0006 (6)	0.0018 (6)

### Geometric parameters (Å, º)

**Table:**

S1—C8	1.7432 (15)	C2—C3	1.382 (2)
S1—C7	1.8161 (17)	C3—C4	1.381 (2)
S2—C8^i^	1.7367 (15)	C3—H3	0.9300
S2—C8	1.7368 (15)	C4—C5	1.392 (2)
Cl1—C2	1.7471 (17)	C4—H4	0.9300
N1—C8	1.301 (2)	C5—C6	1.389 (2)
N1—N1^i^	1.383 (2)	C5—H5	0.9300
C1—C2	1.380 (2)	C6—C7	1.511 (2)
C1—C6	1.395 (2)	C7—H7A	0.9700
C1—H1	0.9300	C7—H7B	0.9700
			
C8—S1—C7	102.72 (7)	C6—C5—C4	120.47 (16)
C8^i^—S2—C8	86.03 (10)	C6—C5—H5	119.8
C8—N1—N1^i^	112.28 (8)	C4—C5—H5	119.8
C2—C1—C6	119.25 (15)	C5—C6—C1	119.31 (15)
C2—C1—H1	120.4	C5—C6—C7	119.64 (15)
C6—C1—H1	120.4	C1—C6—C7	121.02 (14)
C1—C2—C3	121.89 (15)	C6—C7—S1	114.82 (11)
C1—C2—Cl1	119.68 (13)	C6—C7—H7A	108.6
C3—C2—Cl1	118.43 (13)	S1—C7—H7A	108.6
C4—C3—C2	118.87 (15)	C6—C7—H7B	108.6
C4—C3—H3	120.6	S1—C7—H7B	108.6
C2—C3—H3	120.6	H7A—C7—H7B	107.5
C3—C4—C5	120.22 (16)	N1—C8—S2	114.69 (11)
C3—C4—H4	119.9	N1—C8—S1	119.12 (11)
C5—C4—H4	119.9	S2—C8—S1	125.76 (9)
			
C6—C1—C2—C3	0.4 (2)	C5—C6—C7—S1	131.52 (14)
C6—C1—C2—Cl1	179.77 (12)	C1—C6—C7—S1	−50.67 (18)
C1—C2—C3—C4	−0.5 (3)	C8—S1—C7—C6	−69.17 (13)
Cl1—C2—C3—C4	−179.85 (13)	N1^i^—N1—C8—S2	−1.62 (19)
C2—C3—C4—C5	0.1 (3)	N1^i^—N1—C8—S1	171.24 (12)
C3—C4—C5—C6	0.4 (3)	C8^i^—S2—C8—N1	0.62 (7)
C4—C5—C6—C1	−0.5 (2)	C8^i^—S2—C8—S1	−171.70 (14)
C4—C5—C6—C7	177.37 (15)	C7—S1—C8—N1	154.09 (12)
C2—C1—C6—C5	0.1 (2)	C7—S1—C8—S2	−33.91 (11)
C2—C1—C6—C7	−177.73 (14)		

Symmetry code: (i) −*x*, *y*, −*z*+1/2.

### Hydrogen-bond geometry (Å, º)

**Table:**

*D*—H···*A*	*D*—H	H···*A*	*D*···*A*	*D*—H···*A*
C3—H3···N1^ii^	0.93	2.59	3.514 (2)	172

Symmetry code: (ii) *x*+1/2, *y*+1/2, *z*.
